# Advances in CAR-T Cell Therapy: From Structural Design Innovations to Clinical Translation Challenges

**DOI:** 10.7150/jca.134677

**Published:** 2026-09-05

**Authors:** Haixia Gao, Yuan Fang, Pingjing Zhang, Qijun Qian

**Affiliations:** 1 Shanghai Mengchao Cancer Hospital & School of Medicine, Shanghai University, Shanghai 200444, China.; 2 Shanghai Cell Therapy Group Co., Ltd, Shanghai 201805, China.

**Keywords:** CAR-T, CAR design, transfection technology, universal CAR-T, *in vivo* CAR-T generation

## Abstract

Chimeric Antigen Receptor T cells (CAR-T) therapy represents a groundbreaking technology in the field of tumor immunotherapy. This innovative approach involves the genetic engineering of T cells to specifically identify and eradicate tumor cells, and it has been shown to yield remarkable efficacy in treating hematological malignancies. This article provides a comprehensive overview of the core advancements in CAR-T technology, emphasizing the evolution of the five generations of CAR structures. Furthermore, it explores the latest innovations in transfection techniques, highlighting optimizations and comparative advantages of viral and non-viral vectors. The future of CAR-T therapy is poised to focus on several validated translational directions: the development of universal off-the-shelf CAR-T cell products, the design of multi-target and logic-gated intelligent CAR architectures, innovative in vivo genetic reprogramming platforms, and synergistic combinatorial therapeutic regimens to overcome solid tumour immune suppression. These advancements aim to enhance the specificity, safety, and accessibility of CAR-T therapies. This article seeks to outline the current state of CAR-T technology while providing insights into potential theoretical frameworks and technical pathways for its future evolution.

## 1. Introduction

### 1.1 Definition and Technical Foundations of CAR-T Therapy

Chimeric Antigen Receptor T cells (CAR-T) therapy involves genetically engineering T cells to express synthetic chimeric antigen receptors (CARs). This modification enables them to kill tumor cells by targeting surface antigens independently of the major histocompatibility complex (MHC) 1,2. This unique feature of MHC independence enables CAR-T cells to circumvent the downregulation of MHC class I molecules by tumor cells 3,4. Consequently, CAR-T cells can directly engage tumor-associated surface antigens, triggering immediate, potent, and target-specific T cell-mediated cytotoxicity. Decades of preclinical mechanistic validation and global registrational trials have solidified the translational value of MHC-unrestricted CAR targeting, and this core design logic remains the foundational principle for all next-generation CAR platforms developed after 2024 5.

As the clinical and regulatory landscape has rapidly matured, global health agencies have scrutinized and subsequently authorized several highly effective CAR-T therapies. As of 2026, the United States Food and Drug Administration (FDA) and the European Medicines Agency (EMA) have granted commercial approval to seven distinct CAR-T products 6-23. These therapies predominantly target the CD19 and B-cell maturation antigen (BCMA) pathways, providing unprecedented survival benefits for patients suffering from severe B-cell malignancies and relapsed/refractory multiple myeloma. Table 1 provides a highly precise, updated summary of these commercially approved advanced cell therapies, outlining their active biological ingredients, specific target antigens, and current regulatory statuses across major global markets.

Currently, CAR-T clinical trials worldwide focus on two key technological directions: structural optimization and improvements in transfection methodologies 24,25. Structural optimization aims to enhance the therapeutic efficacy of CAR-T products. Meanwhile, innovations in transfection seek to address persistent manufacturing bottlenecks that impede scalability 26. Despite these advancements, the applicability of CAR-T therapy to solid tumors is challenged by issues such as antigen heterogeneity, and the immunosuppressive landscape of the tumor microenvironment (TME) 27,28. Given these challenges, in vivo transfection techniques present a promising alternative by offering a more streamlined approach that sidesteps the complexities often associated with ex vivo methodologies 29,30. This review aims to summarize these key directions in CAR-T research and development, based on a comprehensive review of the relevant literature.

### 1.2 The Comprehensive Framework of CAR-T Technology Development

The core advancements in CAR-T technology encompass structural modularization and a variety of transfection methods. The structural evolution ranges from the fundamental activation mechanisms of the first generation to the sophisticated regulatory capabilities of the fifth generation, while transfection methodologies shift from in vitro viral approaches to in vivo non-viral techniques 31,32. This synergy has propelled significant clinical progress in therapeutic efficacy and safety. Existing literature indicates that iterative engineering strategies effectively tackle T cell exhaustion and reduce cost and risk through enhancements in transfection methodologies 33.

## 2. Modular Design and Intergenerational Evolution of CAR-T Structures

The effectiveness and specificity of CAR-T therapy mainly depend on the structural design of its key component—the CAR 34. This CAR is a synthetically engineered receptor characterized by the seamless integration of the targeting specificity of antibodies and the cytotoxic capabilities inherent to T cells. Over more than three decades, the CAR structure has evolved from a simple signaling module into a complex biological system with sensing, activation, and regulatory functions. Grasping its modular architecture, and the logic behind its iterative evolution, is crucial for understanding the progression of this technology, the refinement of current therapeutic approaches, and the paving of the way for future innovations 35.

### 2.1 Modular Design of CAR

A fully functional CAR molecule can be regarded as a sophisticated machine composed of four core functional modules, each of which significantly influences its ultimate performance. Among these modular components, the extracellular antigen-recognition unit acts as the primary targeting sensor that mediates initial tumor antigen binding. This domain typically consists of a single-chain variable fragment (ScFv), formed by linking the variable heavy chain (VH) and variable light chain (VL) of an antibody with a short peptide 36,37. It is responsible for the specific recognition of target antigens on the surface of tumor cells. The key design consideration lies in balancing affinity and specificity. Excessively high affinity may cause high-density antigen cells to deplete CAR-T cells before they engage with tumor cells expressing low-density antigens. This may also cause CAR-T cells to attack normal tissues with low levels of antigen expression, resulting in on-target/off-tumor toxicity 38. Therefore, screening and optimizing ScFvs using techniques such as phage display serve as the starting point for achieving safe and effective targeting.

While the extracellular domain undertakes antigen capture as the primary sensing unit, the hinge and transmembrane segments act as a critical intermediate mechanical bridge that transmits extracellular binding signals inward and stabilizes CAR membrane localization, making their structural parameters decisive for downstream signal transduction efficiency. The hinge region (or spacer region) connects the ScFv to the transmembrane domain, and its length and spatial flexibility are critical parameters, as sufficient structural freedom is required for ScFvs to access protein-shielded tumour antigen epitopes on cell membranes 39. Studies indicate that an excessively long hinge may increase the risk of nonspecific activation, while a very short hinge could hinder antigen recognition 40. Commonly used hinge regions are derived from molecules such as CD8α and CD28. The transmembrane region anchors the CAR within the T cell membrane and affects its stability and signal transduction. It may mediate dimerization of the CAR molecule with endogenous signaling proteins, such as the CD3 complex, which in turn affects the intensity of T cell activation. Transmembrane domains are often sourced from CD3ζ, CD28, or CD8α 39,41.

Upon stable membrane anchoring mediated by the hinge-transmembrane complex, intracellular signaling domains function as the central execution engine that converts extracellular antigen-binding cues into sustained T cell proliferation and cytotoxic signals, directly determining the in vivo persistence and antitumor potency of engineered CAR-T cells. First-generation CARs contain only the CD3ζ chain, which provides the primary signal (Signal 1) for T cell activation but is insufficient for full T cell activation, leading to limited *in vivo* expansion and persistence. The revolutionary breakthrough of second-generation CARs is the incorporation of a co-stimulatory domain, such as CD28 or 4-1BB, which provides a secondary signal (Signal 2) 42,43. These two domains have distinct functional roles: the CD28 domain tends to drive rapid activation and potent cytotoxicity of CAR-T cells, whereas the 4-1BB domain promotes mitochondrial biogenesis more effectively, enhancing cell survival and long-term persistence 24. This choice directly impacts the characteristics of clinical products. For instance, Yescarta, which targets CD19, employs a CD28 co-stimulatory domain 44, while Kymriah, also targeting CD19, utilizes a 4-1BB co-stimulatory domain 14,45,46.

### 2.2 Generational Evolution: From Linear Enhancement to Functional Expansion

The iterative upgrade of CAR intracellular signalling cassettes from first to fifth generations is not merely structural theory, but has been fully validated by decades of in vitro lymphocyte experiments, murine xenograft models and successive landmark human registrational clinical trials (Table 2. First-generation CAR constructs solely equipped with CD3ζ intracellular motifs laid the original MHC-independent targeting prototype in laboratory settings 1. However, two pioneering phase I solid tumour trials in 2006 delivered definitive clinical proof of their critical translational defects: carbonic anhydrase IX (CAIX)-targeted first CAR-T induced severe on-target renal epithelial toxicity due to unrestricted antigen binding, while folate receptor CAR-T barely expanded in patients and produced zero durable tumour regression 47,48. These early human data confirmed that CD3ζ-only signalling cannot sustain functional T cell responses in vivo, creating an urgent demand for co-stimulatory domain engineering.

The development of second-generation CAR platforms represented a landmark technical breakthrough, resolving the critical limitation of incomplete T cell activation observed in first-generation constructs 49. Two distinct second-generation CAR platforms (CD28 vs 4-1BB backbone) now form the backbone of all seven FDA-approved CAR-T agents, with 3-5 year long-term registrational follow-up data revealing consistent functional disparities across patient cohorts 20,50. CD28-containing CAR-T (axi-cel, ZUMA-1 trial) drives rapid effector T cell proliferation, yielding an 82% objective response rate (ORR) in relapsed DLBCL yet accompanied by 28% grade 3 neurotoxicity at 5-year tracking 20; by contrast, 4-1BB-based tisa-cel in the ELIANA paediatric B-ALL trial generates central memory-biased T cells, with 63% 3-year overall survival and far lower severe neurotoxicity incidence (13%) 51. These real-world clinical datasets directly interpret how co-stimulatory domain selection reshapes CAR-T safety and durability in human patients.

The rationale behind third-generation CARs involved the addition of co-stimulatory signals to enhance effectiveness; however, clinical outcomes have not consistently demonstrated superior efficacy when compared to second-generation CARs. Preclinical murine mesothelioma models validated robust tumour clearance for CD28/4-1BB dual-signalling third-generation CARs 52; nevertheless, the first within-patient head-to-head phase I human trial published in 2026 generated mixed clinical readouts. Although third-generation CARs exhibited higher early peripheral expansion in subjects, no statistically significant improvement in long-term progression-free survival was observed compared to second-generation counterparts, while the risk of high-grade Cytokine Release Syndrome (CRS) was moderately elevated 52. This direct human comparative trial resolves the discrepancy between promising preclinical performance and ambiguous clinical benefit for multi-costimulatory CAR designs. This observation indicates that simply augmenting signal strength may not be the optimal strategy, but maintaining a balance in T cell functionality and preventing exhaustion could be more crucial than achieving absolute signal intensity 53.

While signal tuning dominated early structural iterations, later CAR generations delivered a fundamental paradigm shift, transforming engineered lymphocytes from simple tumor-killing effectors into multifunctional immune regulatory platforms capable of remodeling the hostile tumor microenvironment. The fourth-generation CAR (TRUCK) represents a significant step forward in design philosophy. It goes beyond the traditional role of CAR-T cells as cytotoxic agents. Instead, it re-engineers them into “bioreactors” that can reconfigure the TME 54. For example, by utilizing NFAT response elements, CAR recognition of tumor antigens induces IL-12 expression, which reverses the immunosuppressive environment of the TME and recruits macrophages, natural killer (NK) cells, and other immune players to collaborate against the tumor. This is particularly important in addressing the challenge of antigen heterogeneity in solid tumors. The fifth-generation CAR endeavors to integrate the intracellular domains of cytokine receptors (such as IL-2Rβ), with the aim of concurrently receiving antigen-specific signals and cytokine signals, providing a more nuanced integration and amplification of endogenous signals; however, this approach remains in the exploratory phase 55.

## 3. Innovations and Challenges in Vectors and Transfection Technologies

The clinical translation of CAR-T therapy is significantly dependent on effective and safe gene delivery technologies. An ideal vector system needs to strike a balance among several critical factors, including transfection efficiency, cell viability, risks associated with genomic integration, production costs, and adherence to clinical regulations. In recent years, vector technologies and transfection methods have transitioned from a predominantly viral vector-based approach to a more diverse range that encompasses viral systems, non-viral systems, and emerging *in vivo* programming techniques. Notably, advancements in in vivo delivery technologies are driving the transformation of CAR-T therapy from “factory-based manufacturing” to “in situ reprogramming” 29,57. This shift seeks to address key challenges of traditional therapies, including long treatment cycles, high costs, and limited accessibility 58.

### 3.1 Innovations in Vector Technology

Vectors serve as “vehicles” for CAR genes, with their design directly impacting delivery efficiency and safety. They are broadly categorized into viral and non-viral types (Table 3). While lentivirus and γ-retroviruses dominate current approved CAR products, a wave of non-viral delivery systems have matured over the past two years to address viral vector-associated cost and safety drawbacks, with transposon and lipid nanoparticle systems standing out as the most translationally promising candidates 34,59.

Viral vectors, particularly lentiviral vectors, continue to be the primary delivery tools for current clinical CAR-T products. These vectors achieve efficient infection through modifications to the viral envelope (for instance, by utilizing vesicular stomatitis virus glycoprotein (VSV-G)). However, their random integration characteristics present a potential risk for insertional mutations 70. To mitigate this issue, next-generation engineered lentiviral vectors are focusing on “targeted” modifications, such as altering the viral envelope to specifically recognize T cell surface antigens (like CD3), thereby reducing the risk of off-target effects 71. Beyond envelope modification, multi-layer backbone and integration optimizations further improve vector safety and manufacturability: fourth-generation TetraVecta lentiviral backbones delete redundant accessory genes to suppress innate immune responses during good manufacturing practice (GMP) culture 56; capsid engineering via peptide libraries and glycan remodeling resolves prevalent human anti-AAV neutralizing antibodies 72; CRISPR-directed CAR insertion at TRAC/PDCD1 safe harbors fundamentally eliminates oncogenic integration near proto-oncogenes 73.

To bypass the persistent genotoxic and manufacturing burdens of viral platforms, researchers have vigorously advanced non-viral delivery systems with integration-free safety profiles, among which LNPs stand out as the most translationally mature candidate. LNPs play a central role in mRNA delivery, capitalizing on the advancements in mRNA vaccine technology and showcasing strong clinical translation potential 71. For example, Capstan Therapeutics employs targeted LNPs (tLNPs) to deliver CD19 CAR mRNA directly to CD8+ T cells in vivo for the treatment of autoimmune diseases 70. Plasmid DNA, the simplest form of gene delivery, still retains its value in basic research and preclinical tool development; However, due to its lower transfection efficiency and challenges with stable integration, it is likely to be gradually supplanted by more efficient technologies in clinical applications 56,72,73. Recent GMP-ready transposon platforms have advanced to early-phase human trials: Sleeping Beauty-based TranspoCART19 equipped with iCasp9 suicide switches is undergoing Phase I assessment for B-cell malignancies, whereas PiggyBac CAR constructs targeting solid tumors deliver simplified, low-cost plasmid manufacturing pipelines. Hybrid LNP-transposon dual delivery systems combining transposase mRNA and CAR plasmids achieve stable genomic integration without viral reagents, and circular mRNA-LNP vehicles outperform linear mRNA in prolonging CAR expression and alleviating type I interferon immune stimulation. Notably, advances in protein engineering have yielded multiple hyper-active transposase variants: SB100X for Sleeping Beauty, hyPBase for PiggyBac, and BZ-series mutants engineered from the zebrafish-derived ZB transposon (Tc1/mariner superfamily). These protein-optimized enzymes substantially raise transposition efficiency and permit tighter tuning of transgene copy number, accelerating the clinical translation of transposon-derived CAR-T products 66,67,68.

### 3.2 Diverse Development of Transfection Technology

Transfection technology refers to the methods used to introduce exogenous genes into cells, which can be classified into chemical methods, physical methods, and biological methods (such as viral transduction). The selection of the appropriate method should consider factors such as the type of cells involved, the specific objectives of the experiment, and the context of clinical applications 74. The traditional *in vitro* transfection techniques have been continuously refined, with electroporation still serving as a commonly employed method for the transfection of primary T cells.

While incremental improvements have been made to ex vivo electroporation and viral transduction pipelines, their inherent drawbacks including mandatory leukapheresis and weeks-long manufacturing cycles severely limit widespread clinical deployment, thereby catalyzing the disruptive innovation of in vivo CAR reprogramming platforms 71. This innovative approach focuses on the direct delivery of CAR gene vectors (such as lipid nanoparticles or viral vectors) into the patient's body via systemic administration methods, such as intravenous injection, facilitating the generation of CARs within T cells *in vivo* and eliminating the need for all external manipulation steps (Table 4) 75. As a preliminary human exploration of such mRNA-LNP in vivo CAR platforms, a 2025 clinical research report published in New England Journal of Medicine described a single-arm exploratory trial applying mRNA-LNP-based in vivo CD19 CAR-T for refractory systemic lupus erythematosus (SLE), enrolling only 5 patients 76. This trial captured transient CAR expression in circulating T cells and provided preliminary evidence of acceptable short-term safety profiles. However, definitive therapeutic efficacy and long-term safety remain unsubstantiated due to the extremely limited sample size. Larger, randomized controlled phase III trials with extended follow-up are still required to confirm the clinical value of this in vivo CAR strategy for SLE treatment.

### 3.3 Breakthroughs and Challenges in *In Vivo* Programming

*In vivo* CAR-T generation technology is currently at the cutting edge, aiming to directly reprogram T cells within patients through a single injection of a gene delivery vector, enabling them to express CAR 57. The central challenge lies in achieving efficient and specific in vivo targeted delivery.

LNP-mRNA Pathway: This pathway delivers CAR mRNA by designing LNPs that specifically target T cells (for example, using surface-conjugated anti-CD8 antibodies). Its primary advantage is high safety, as the mRNA is only expressed transiently in the cytoplasm, does not integrate into the genome, and has controllable side effects 29,78,79,80. For instance, if adverse reactions occur, CAR proteins can be rapidly cleared after medication is discontinued. This 2025 NEJM Phase I cohort enrolled five heavily pretreated refractory lupus patients treated without prior lymphodepletion chemotherapy and achieved rapid peripheral B cell depletion. Nevertheless, mRNA-mediated CAR expression only lasts 7-10 days and results in gradual autoreactive B cell recovery. Preclinical CD5-targeted LNPs loaded with FAP CAR mRNA expand the therapeutic scope to fibrotic diseases, while Capstan and Interius' in vivo LNP programs have entered Phase I trials; systemic hepatic sequestration and inconsistent endogenous T cell counts remain core translational barriers.

Beyond targeting-efficiency bottlenecks, in-vivo CAR-T systems carry unique safety risks that do not exist for conventional ex-vivo-manufactured CAR-T products 75. Systemically infused delivery vehicles (tLNPs or targeted viral vectors) may produce off-target transfection of non-T-cell populations, including myeloid cells and hepatocytes, which could drive aberrant CAR expression in unintended cell types and trigger unforeseen local inflammation or antigen-directed cytotoxicity 77. For LNP-based platforms, prominent hepatic sequestration represents a well-documented translational risk; substantial fractions of administered nanoparticles are scavenged by liver-resident macrophages, potentially elevating liver enzyme levels and innate immune cascades upon repeated dosing 79. Multiple complementary mitigation approaches are being actively investigated to reduce these hazards. First, surface engineering of delivery carriers (incorporating anti-CD3/CD8 targeting moieties, fusogen modification for viral vectors) improves T-cell-selective tropism and reduces uptake by off-target cell subsets. Second, employing non-integrating mRNA payloads delivers only transient CAR expression; even if off-target transfection occurs, CAR protein will degrade within days without genomic modification, limiting persistent adverse consequences. Third, orthogonal safety-control modules such as inducible suicide switches (e.g., iCasp9) or small-molecule-regulated ON/OFF CAR circuits can be co-incorporated into vector constructs, enabling rapid, pharmacologic ablation of CAR-bearing cells should toxic manifestations emerge 75. Even with these safeguards, thorough pre-clinical biodistribution profiling and careful monitoring of liver function, cytokine levels, and off-tissue CAR-positive cell populations remain mandatory for early-phase clinical trials of systemically administered in-vivo CAR-T therapeutics.

However, *in vivo* programming technology still encounters several significant challenges. These challenges include bottlenecks in targeting precision and efficiency, as ensuring that the vector only infects the intended T cell subpopulation without damaging other cells is fundamental to safety. A key technical barrier is how to effectively concentrate the systemically administered vector in the target T cells, rather than having it captured by the liver or other tissues 81. Additionally, individual variability poses a major challenge, as there are significant differences in both the quantity and condition of T cells among patients, making it difficult to establish a universal dosing regimen.

## 4. Challenges in CAR-T Technology

Despite the significant achievements of CAR-T therapy in the treatment of hematologic malignancies, its further development and widespread application still face multiple severe challenges, primarily in three areas: safety, effectiveness in treating solid tumors, and production processes and accessibility 31,82.

### 4.1 Safety Challenges: Toxic Side Effects and Management

CAR-T cell therapy presents significant safety risks due to excessive activation and nonspecific killing of cells, primarily manifesting as CRS and Immune Effector Cell-Associated Neurotoxicity Syndrome (ICANS) 83. The underlying mechanism involves the recognition of target antigens by CAR-T cells, which leads to their massive proliferation and the release of inflammatory cytokines (such as IL-6 and IFN-γ) 84. These responses can cause severe symptoms such as high fever, hypotension, and hypoxia, which may be life-threatening. Additionally, neurotoxicity may present as confusion, dysphasia, or cerebral edema. Tocilizumab (an anti-IL-6 receptor antibody) and corticosteroids are currently the first-line therapies used to treat CRS 85. A more profound concern is the potential risk of secondary T-cell malignancies. The U.S. FDA has issued black box warnings for all marketed BCMA- and CD19-targeted CAR-T products to flag a potential correlative link to secondary T-cell malignancies, yet the exact causative pathways remain incompletely defined. Random genomic integration of lentiviral vectors is a widely hypothesized contributing mechanism, as vector insertion near endogenous proto-oncogenes may theoretically drive oncogene overexpression; however, this integration event cannot be confirmed as the sole definitive causal factor, with multiple pre-existing patient clonal hematopoiesis and prior chemotherapy co-variables jointly implicated 86. According to 2024 FAERS pharmacovigilance database analysis, secondary T-cell malignancy case submissions account for approximately 0.1% of all CAR-T-associated adverse event records submitted to the FDA. Despite the low proportional volume of these adverse event submissions, long-term longitudinal safety follow-up remains mandatory for all CAR-T recipients per FDA regulatory guidance to monitor rare late-onset oncogenic complications 87.

Additionally, off-target and off-tumor toxicity pose significant issues, as CAR-T cells may attack healthy organs when the target antigen is expressed at low levels on normal tissues. To address these risks, innovative safety strategies are actively being explored, such as inducible suicide genes (like the iCasp9 system) and logic-gated designs (such as AND-gate CAR-T).

### 4.2 Multiple Barriers to the Treatment of Solid Tumors

Hematological malignancies and solid tumours exhibit drastically divergent biological landscapes that generate stark discrepancies in CAR-T clinical outcomes. A pooled systematic analysis of phase I/II clinical data spanning the past five years reveals a pronounced therapeutic dichotomy: CD19/BCMA-targeted CAR-T yields objective response rates (ORRs ranging 70%-90%) with sustained complete remission in relapsed B-cell neoplasms, while solid tumour CAR trials rarely achieve ORRs exceeding 10%-20%, with most clinical benefits limited to transient disease stabilization rather than curative tumour eradication 88,89. Table 5 systematically quantifies and contrasts core clinical and biological disparities between the two tumour categories.

To overcome these interconnected, multifaceted solid tumour barriers, layered engineered and combinatorial therapeutic strategies validated by abundant preclinical and early human trial datasets have been developed 28,44. First, to address antigen heterogeneity, dual- or multi-target CAR designs (e.g., targeting both EGFRvIII and IL13Rα2 in glioblastoma, or Claudin18.2 and MUC1 in gastric cancer) are utilized to prevent immune escape driven by antigen shedding or natural downregulation. 88,91,92. Second, intrinsic T cell functional enhancement is achieved by engineering CAR-T cells to co-express extracellular matrix (ECM)-degrading enzymes, such as heparanase, enabling the cells to physically drill through the fibrotic stroma to reach the tumor core. Finally, combinatorial therapeutic regimens are widely considered paramount for solid tumor success; co-administering CAR-T cells with immune checkpoint inhibitors (e.g., anti-PD-1 or anti-PD-L1 monoclonal antibodies) serves to block the terminal inhibitory pathways that trigger T cell exhaustion immediately upon entry into the highly suppressive TME 28,93,94,95.

### 4.3 Manufacturing, Quality Control, and Economic Translation Challenges

The complex individualized manufacturing workflow, stringent global regulatory quality control standards, and prohibitive production costs collectively constitute a core category of clinical translational barriers for CAR-T therapeutics. Autologous CAR-T relies on patient-derived lymphocytes for one-off bespoke production, a characteristic that creates inherent batch variability, long treatment waiting periods and severe accessibility disparities worldwide 96. The complete vein-to-vein manufacturing pipeline consists of sequential leukapheresis for T cell collection, cryopreserved cold-chain transportation to centralized GMP facilities, T cell enrichment and activation, viral vector-mediated genetic transduction, bioreactor expansion, multi-layer quality control release testing, and final shipment back to clinical wards for patient infusion, with a standard production cycle spanning 3-6 weeks per individual patient product 97. This lengthy workflow often leads to disease progression in late-stage tumour candidates before CAR-T preparation is completed, disqualifying many patients from receiving curative cell therapy.

Unlike small-molecule or antibody drugs with unified industrial production lines, CAR-T as a living cellular medicinal product requires highly rigorous lot release testing defined as critical quality attributes (CQAs) under FDA and EMA regulatory frameworks. Conventional pharmaceutical detection systems are often insufficient to evaluate its safety and functional activity. Four core mandatory quality control (QC) testing modules are enforced for final batch approval before clinical infusion 98,99:

1. Microbial safety and viral risk verification: Each finished product undergoes comprehensive sterility testing, mycoplasma and endotoxin quantification, alongside high-sensitivity molecular detection to eliminate replication-competent lentivirus (RCL) or retrovirus contaminants that carry life-threatening insertional mutagenesis risks.

2. Cell identity and purity profiling: High-parameter flow cytometry confirms a high proportion of CD3+ T cells (e.g., ≥90%), balanced CD4/CD8 subset ratios, and removal of residual malignant tumor cells and magnetic activation microbead impurities.

3. Functional potency quantification: Calibrated co-culture cytotoxicity assays measure specific tumor lysis capacity and antigen-triggered secretion of effector cytokines (e.g., IFN-γ and TNF-α), serving as a core readout of in vivo therapeutic potential.

4. Genomic stability assessment: qPCR vector copy number (VCN) detection is used to ensure a limited number of integrated vector copies per T cell (e.g., fewer than 4-5), balancing sufficient CAR surface expression with the risk of oncogenic gene insertion. All QC assays must be completed within a narrow post-expansion time window, as live CAR-T cells cannot be stably cryopreserved for repeated retesting.

The culmination of these bespoke manufacturing steps, highly skilled labor requirements, and exhaustive QC assays drives the cost of ex vivo CAR-T therapy to extraordinary, highly prohibitive heights. In Western markets, baseline commercial costs generally range from $373,000 to over $500,000 per dose, completely excluding the ancillary costs of hospitalization, critical care for toxicity management, and long-term follow-up 100. This economic reality restricts access primarily to elite healthcare systems in high-income countries.

To counteract this profound global health inequality, alternative manufacturing and economic paradigms are being aggressively explored. An emergent, highly successful strategy involves localizing production in emerging markets or utilizing decentralized, point-of-care manufacturing at specific academic hospitals. A landmark achievement in this arena occurred recently when India approved its first indigenous CAR-T product, NexCAR19 (actalycel) 101. Developed through a collaboration between ImmunoACT, the Indian Institute of Technology (IIT) Bombay, and Tata Memorial Hospital, NexCAR19 was engineered specifically for resource-constrained environments. By establishing independent, domestic viral vector manufacturing and streamlining the entire supply chain through a hub-and-spoke model, the total cost of NexCAR19 was reduced to approximately $30,000 to $50,000. This represents a staggering 10-fold reduction compared to standard global pricing models, unequivocally demonstrating that localized biomedical innovation and academic-industry partnerships can drastically improve global patient accessibility to advanced cellular therapies.

## 5. Future Development Trends and Innovative Directions

To overcome the aforementioned bottlenecks, CAR-T technology is rapidly evolving toward safer, more efficient, and more accessible solutions, giving rise to several promising innovative directions.

Universal CAR-T, also known as “off-the-shelf” CAR-T, aims to utilize T cells from healthy donors for large-scale, standardized production, creating readily available “off-the-shelf” products 102. The core technology involves employing gene editing techniques (such as CRISPR/Cas9) to knock out the T cell receptor (TCR) and HLA class I molecules in donor T cells, thereby avoiding graft-versus-host disease (GvHD) and host immune rejection 103. Although challenges remain regarding in vivo persistence, universal CAR-T holds the potential to significantly reduce costs and preparation timelines, making it a key pathway to address accessibility issues 102. Several companies, including Cellectis and Allogene, are actively investing in this area.

*In Vivo* CAR-T generation technology represents one of the most disruptive frontiers, aiming to achieve “in situ reprogramming” by intravenously delivering a targeted delivery system (such as lipid nanoparticles or viral vectors) that directly introduces the gene encoding CAR (DNA or mRNA) into the patient's T cells. This technology eliminates all steps of ex vivo manufacturing, transforming CAR-T therapy from a “cell product” into a “gene therapy,” thereby greatly simplifying the process and reducing costs 57. A groundbreaking 2025 study demonstrated that in vivo generation of transient FAP-targeting CAR-T cells can be achieved through a single intravenous injection of CD5 antibody-directed LNPs encapsulating CAR mRNA. This approach successfully reversed established pulmonary fibrosis and promoted lung regeneration in mouse models, highlighting the platform's immense therapeutic potential for treating fibrotic diseases 104. Companies such as Capstan Therapeutics and Interius BioTherapeutics are benchmark enterprises in this field, and their INT2104 and CPTX-2309 clinical programs are actively exploring the clinical application of this technology 75.

To enhance safety and combat solid tumors, the next generation of CAR-T designs focuses on “intelligence” and functional integration. This includes: Logic-gated systems, such as AND and NOT gates, which require multiple conditions to be met for CAR-T cell activation, allowing precise differentiation between tumor and normal tissues 35,105. Armored CAR-T: By co-expressing cytokines (such as IL-12, IL-15) or chemokines, this design modifies and reshapes the immunosuppressive tumor microenvironment, enhancing CAR-T cell functionality and recruiting endogenous immune cells 106. Tunable systems: Introducing “molecular switches” that can be controlled by small molecules (such as rapamycin or tetracycline) enables real-time precise regulation of CAR-T cell activity 107.

Beyond CD19 and BCMA, researchers are actively developing new targets for solid tumors—such as Claudin18.2, GPC3, and B7-H3—as well as dual- and multi-target CAR-T therapies, such as CD19/CD22, to address antigen escape and heterogeneity 91,92. The combination of CAR-T with other therapies demonstrates synergistic potential. For instance, combining with immune checkpoint inhibitors (such as PD-1/PD-L1 antibodies) can reverse T cell exhaustion 94; combining with oncolytic viruses can degrade solid tumor barriers and amplify antitumor effects 95; and combining with targeted therapies or radiotherapy can enhance antigen release and immune responses 108.

## 6. Conclusion

CAR-T cell therapy is experiencing a profound technological transformation. In the next five to ten years, collaborative innovations in areas such as universal technology, in vivo programming, and intelligent design are expected to significantly improve the safety, efficacy, and accessibility of CAR-T therapy. This progress will transform CAR-T therapy from a highly customized “elite therapy” into a safer, more effective, and affordable treatment accessible to a wider range of cancer patients and individuals with autoimmune diseases.

## Figures and Tables

**Figure 1:**
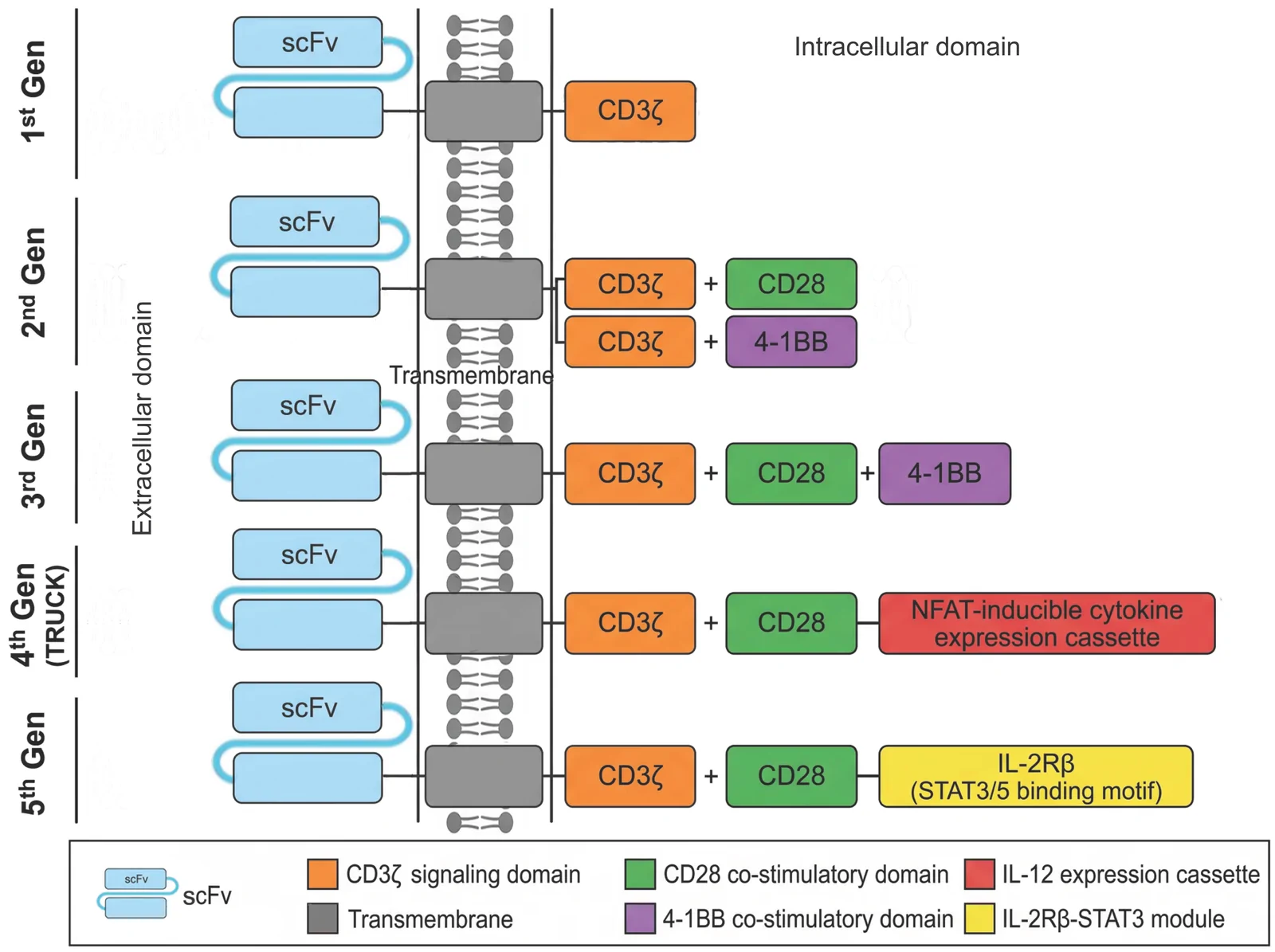
** Structural comparison of 1st to 5th-generation CAR molecules.** Schematic overview of progressive modular upgrades of CAR constructs anchored on the T cell membrane. The signalling capacity evolves from the single CD3ζ domain in 1st-generation CARs, addition of single/dual co-stimulatory domains in 2nd/3rd-generation CARs, integration of cytokine expression cassettes in 4th-generation TRUCK CARs, and incorporation of IL-2Rβ (STAT3/5) signalling modules in 5th-generation CARs.

**Table 1:** FDA and EMA Approved CAR-T Cell Therapies

Commercial Name	Active Ingredient (INN)	Target Antigen	Primary Clinical Indication	Regulatory Approval Status
Abecma	Idecabtagene vicleucel	BCMA	Relapsed/Refractory Multiple Myeloma (RRMM)	FDA Approved (2021), EMA Approved 6,7
Aucatzyl	Obecabtagene autoleucel	CD19	Relapsed/Refractory B-cell Precursor Acute Lymphoblastic Leukemia (B-ALL) in adults	FDA Approved (Nov 2024), EMA Approved (July 2025) 8
Breyanzi	Lisocabtagene maraleucel	CD19	Large B-cell Lymphoma (LBCL), Follicular Lymphoma	FDA Approved (2021), EMA Approved 9-11
Carvykti	Ciltacabtagene autoleucel	BCMA	Relapsed/Refractory Multiple Myeloma (RRMM)	FDA Approved (2022), EMA Approved 12,13
Kymriah	Tisagenlecleucel	CD19	B-ALL (up to age 25), LBCL, Follicular Lymphoma	FDA Approved (2017), EMA Approved 14-16
Tecartus	Brexucabtagene autoleucel	CD19	Mantle Cell Lymphoma (MCL), Adult B-ALL	FDA Approved (2020), EMA Approved 17,18
Yescarta	Axicabtagene ciloleucel	CD19	LBCL, Follicular Lymphoma	FDA Approved (2017), EMA Approved 19-21

No post-market data requiring further elaboration were identified for Aucatzyl since its EMA approval in 2025. The table reflects the approved information at the time of writing.

**Table 2:** Structural Characteristics and Functional Evolution of CARs Across Generations (Figure 1)

Generation	Structural Features	Functional Evolution	Advantages	Disadvantages
1st Generation	- Single-chain variable fragment (ScFv) targeting antigen - CD3ζ intracellular domain for T cell activation 3,56	- Basic activation of T cells - Low cytotoxicity and limited antitumor efficacy	Simple design, initial proof of concept for CAR-T therapy.	Limited efficacy, lack of co-stimulatory signals leading to poor T cell activation and persistence.
2nd Generation	- ScFv targeting antigen - CD3ζ domain + co-stimulatory domain (e.g., CD28 or 4-1BB) 42,43	- Enhanced T cell activation and proliferation - Improved antitumor response	Improved T cell activation and proliferation, enhanced antitumor efficacy compared to 1st generation.	Still limited in functionality, potential for over-activation leading to toxicity.
3rd Generation	- ScFv targeting antigen - CD3ζ domain + dual co-stimulatory domains (e.g., CD28 and 4-1BB) 52	- Further improved T cell activation - Increased persistence and efficacy against tumors	Further enhanced T cell activation, better persistence and efficacy in tumor targeting.	Increased complexity, potential for increased side effects and management challenges.
4th Generation	- ScFv targeting antigen - CD3ζ domain + co-stimulatory domains + additional transgenic elements (e.g., cytokines) 54	- Inducible release of immune modifiers (e.g., IL-12) - Modulation of tumor microenvironment	Greater control over T cell activity, improved antitumor responses, potential for combination therapies.	Complexity in design and manufacturing, risk of cytokine release syndrome.
5th Generation	- ScFv targeting antigen - CD3ζ domain + co-stimulatory domain + truncated IL-2Rβ intracellular domain containing STAT3/5 binding motif to trigger endogenous JAK-STAT signaling 55	- Integration of cytokine receptor signal cascade to drive homeostatic T cell proliferation and stemness maintenance Dual antigen targeting can be optionally added as auxiliary modification	Sustained T cell persistence and stem-like memory phenotype via autonomous JAK/STAT cytokine signaling; retains flexible multi-targeted design as optional modification.	Complex vector construction; long-term in vivo safety of constitutive cytokine receptor signaling remains under further clinical validation.

CAR: chimeric antigen receptor

**Table 3:** Comparison of CAR-T Vector Technologies

Category	Specific Vector Platform	Mechanism of Action	Advantages	Disadvantages & Challenges
Viral Vectors	Lentivirus (LV) 60,71	Infects dividing and non-dividing cells. Its RNA genome is reverse-transcribed into DNA and randomly integrated into the host genome, enabling long-term transgene expression.	• High Transduction Efficiency: Highly effective for gene delivery into primary human T cells. • Stable Long-term Expression: Genomic integration ensures CAR persistence, which is critical for durable clinical responses. • Clinically Validated: The current gold standard for most approved ex vivo CAR-T therapies.	• Risk of Insertional Mutagenesis: Random integration poses a theoretical risk of oncogene activation (prompting an FDA black box warning). • Complex and Costly good manufacturing practice (GMP) Manufacturing: Production is time-consuming and expensive. • Immunogenicity: Pre-existing or developed immunity can neutralize the virus.
Viral Vectors	γ-Retrovirus 61,62	Infects primarily dividing cells. Its RNA genome is reverse-transcribed and randomly integrated into the host chromosome.	• Stable Long-term Expression: Proven ability to generate persistent CAR-T cells. • Historical Precedent: Used in foundational CAR-T clinical trials.	• Higher Risk of Insertional Mutagenesis: Greater propensity for integration near gene promoters compared to LV. • Limited to Dividing Cells: Requires pre-activation/expansion of T cells before transduction. • Largely Superseded by lentivirus in modern clinical development.
Viral Vectors	Adeno-Associated Virus (AAV) 63	Infects non-dividing cells. Its single-stranded DNA genome resides predominantly as non-integrated nuclear episomes, supporting sustained intracellular transgene expression that diminishes gradually over time.	• Excellent In Vivo Delivery: Well-established platform for efficient in vivo gene delivery to tissues. • Favorable Safety Profile: Low pathogenicity and primarily non-integrating.	• Small Cargo Capacity (~4.7 kb), often too small for complex CAR constructs. • High Prevalence of Pre-existing Immunity: Neutralizing antibodies in a large population can inactivate the vector. • Not Ideal for Ex Vivo CAR-T: Primarily explored for in vivo delivery due to cost and immunogenicity hurdles.
Non-Viral Vectors	Plasmid DNA 64	Circular DNA vectors are delivered into the cell nucleus via electroporation. The plasmid persists as a non-integrating episome, with expression diluted through cell division.	• Simple and Inexpensive Production: Easy to design and manufacture at scale. • Predominantly episomal with negligible genomic integration potential; rare random integration events cannot be fully ruled out. • Large Cargo Capacity.	• Very Low Transfection Efficiency: Highly inefficient in primary T cells. • Transient and Unstable Expression: The plasmid is easily lost, making it unsuitable for lasting therapies. • High Cytotoxicity: The required electroporation severely impacts T cell fitness and viability.
Non-Viral Vectors	mRNA 65	Synthetic mRNA is delivered directly into the cell cytoplasm (via electroporation or nanoparticles) and is translated into protein without nuclear entry or genomic integration.	• High Safety Profile: No risk of insertional mutagenesis; expression is transient (days), allowing for toxicity management. • Rapid Protein Production: CAR expression begins within hours. • Simpler and Cheaper Manufacturing: Avoids the complexities of viral vector production.	• Transient Expression: Requires repeated administrations for sustained antitumor activity. • Cytotoxicity and Immunogenicity: Electroporation reduces cell fitness; mRNA can activate innate immune pathways.
Non-Viral Vectors	Transposon Systems (e.g., Sleeping Beauty, PiggyBac) 64,66, 67,68	A DNA plasmid containing the CAR gene flanked by transposon sequences is co-delivered with transposase mRNA/protein. The transposase facilitates “cut-and-paste” genomic integration of the transgene. Modern hyper-engineered transposase variants include SB100X (Sleeping Beauty), hyPBase (PiggyBac), and BZ-series mutants derived from the zebrafish-origin ZB transposon (Tc1/mariner superfamily); these variants greatly improve transposition efficiency and support tunable transgene copy-number regulation	• Non-Viral Integration: Enables stable, long-term CAR expression without using a virus. • Lower Manufacturing Cost: Simpler and more scalable GMP production than viral systems. • Large Cargo Capacity. • Greatly improved integration performance enabled by recently optimized hyper-active transposase orthologs	• Variable Integration Efficiency: Often lower than viral transduction, though substantially elevated by hyper-active transposase engineering. • Residual Mutagenesis Risk: Integration is not perfectly targeted, though profiles may be safer than random viral integration. • Technical Complexity: Requires optimization of DNA and transposase delivery.
Non-Viral Vectors	Targeted Lipid Nanoparticles (tLNP) 69	Synthetic nanoparticles encapsulating CAR-encoding mRNA or DNA. The LNP surface is functionalized with targeting ligands (e.g., antibodies) to direct it to specific cell types (e.g., T cells) following systemic intravenous infusion.	• *In Vivo* CAR-T Generation: Aims to transform CAR-T therapy from a cell product into an injectable “gene drug.” • Targeted Delivery: Reduces off-target effects and improves specificity. • High Safety Potential (with mRNA): Combines the safety of transient expression with targeted delivery.	• Nascent Technology: Still in early-stage clinical trials; human efficacy is not yet fully established. • Delivery Challenges: Achieving high specificity and efficiency in delivering to the correct T-cell subset in vivo is a major hurdle. • Immune Activation: LNPs and their payload can trigger innate immune responses.

CAR-T: chimeric antigen receptor T-cell.

**Table 4:** Comparison of *Ex Vivo* and *In Vivo* CAR-T Cell Therapy Manufacturing Strategies 77.

Feature	*Ex Vivo* CAR-T Therapy (Conventional)	*In Vivo* CAR-T Therapy (Emerging)
Core Principle & Manufacturing Site	T cells are collected from the patient via leukapheresis, genetically modified, and expanded outside the body (in a centralized GMP facility) before being reinfused.	Genetic instructions for the CAR are delivered directly to the patient's T cells inside the body via an injectable vector.
Cell Sourcing & Logistics	Autologous: Relies on the patient's own cells. Requires complex cold chain logistics for transporting cells to and from the manufacturing facility.	Uses the body's endogenous T cells. Eliminates the need for cell extraction and transport, simplifying logistics.
Genetic Delivery Vector	Primarily uses viral vectors (e.g., lentivirus) for stable genomic integration of the CAR gene in the lab.	Uses viral vectors (lentivirus) or non-viral vectors like targeted LNPs carrying mRNA or other nucleic acid payloads.
Vein-to-Vein Time & Readiness	Lengthy process (2-7 weeks), leading to a significant waiting period. Not an off-the-shelf product.	Rapid administration. Aims for “on-demand” treatment. Designed as an off-the-shelf, readily available product.
Patient Preconditioning	Requires lymphodepleting chemotherapy (e.g., fludarabine/cyclophosphamide) prior to infusion to enhance engraftment.	Potentially eliminates the need for lymphodepletion, as it does not require making “space” for externally expanded cells.
Cost Structure	Extremely high (approx. $500,000 per dose) due to personalized, complex manufacturing and quality control. 34	The elimination of patient-specific ex vivo cell manufacturing provides broad theoretical potential to drastically cut overall therapeutic expenditures; multiple reviews predict substantial long-term cost savings compared to current autologous CAR-T platforms.
Persistence of CAR-T Cells	Long-lasting or permanent due to genomic integration by viral vectors, aiming for durable remission.	Can be transient (especially with mRNA/LNP platforms, lasting days) or persistent (with integrating viral vectors), allowing for dose-tunable activity.
Key Advantages	Established, proven efficacy in hematologic malignancies; controlled manufacturing process.	Simplified administration, lower cost potential, broader accessibility, faster treatment initiation, potential for improved safety profile.
Major Challenges	High cost, complex logistics, long manufacturing time, need for specialized centers, risk of severe toxicities (CRS, ICANS).	Targeting specificity (ensuring CAR gene is delivered only to intended T cells), managing immune responses to the vector, optimizing efficiency, and long-term safety concerns like insertional mutagenesis (for viral vectors).
Representative Companies/Developers	Novartis, Gilead, Bristol Myers Squibb, Johnson & Johnson.	Capstan Therapeutics, EsoBiotec (acquired by AstraZeneca), Umoja Biopharma.
Clinical Stage	Approved and commercially available for several hematologic cancers.	Early-stage clinical trials (Phase I). Primarily in preclinical and discovery phases for many assets.

CAR-T: chimeric antigen receptor T-cell; CRS: cytokine release syndrome; ICANS: immune effector cell-associated neurotoxicity syndrome.

**Table 5:** Systematic Comparison of CAR-T Efficacy: Hematological Malignancies vs. Solid Tumors 89,90.

Clinical / Biological Parameter	Hematological Malignancies (e.g., B-ALL, LBCL, MM)	Solid Tumors (e.g., Glioblastoma, Pancreatic, Gastric Cancers)
Objective Response Rate (ORR)	Exceptionally high (70% - 90%), frequently leading to complete, durable remission.	Generally low (<20%), with clinical benefits mostly limited to transient stable disease.
Antigen Expression Profile	Highly homogeneous and lineage-specific (e.g., CD19 is universally expressed on all B cells).	Extremely heterogeneous; antigen-negative or low-expressing tumor variants frequently drive immune escape.
Trafficking and Infiltration	Excellent access via systemic blood circulation and highly permissive lymphatic tissues.	Severely restricted by highly abnormal tumor vasculature and dense, fibrotic extracellular matrix (ECM).
Tumor Microenvironment (TME)	Relatively permissive to immune effector functions.	Deeply immunosuppressive; characterized by high concentrations of TGF-β, Tregs, tumor-associated macrophages (TAMs), and myeloid-derived suppressor cells (MDSCs).
CAR-T Activation and Longevity	High activation efficiency; potential for years-long central memory persistence.	Rapid metabolic exhaustion driven by chronic, relentless antigen exposure and extreme nutrient starvation (hypoxia).

## Data Availability

All data, analytical frameworks, and structural comparative analyses synthesized and discussed within this manuscript are sourced entirely from publicly accessible peer-reviewed literature, global clinical trial registries, and official regulatory databases (FDA, EMA). No original primary datasets were generated during the composition of this comprehensive review. Specific data points regarding clinical trials, product approvals, health economics, and adverse event incidence rates can be directly accessed via the cited pharmacovigilance reports and published literature identifiers provided throughout the text.

## Abbreviations

AAV: adeno-associated virus
CAIX: carbonic anhydrase IX
CAR: chimeric antigen receptor
CAR-T: chimeric antigen receptor T-cell
CQAs: critical quality attributes
CRS: cytokine release syndrome
ECM: extracellular matrix
FDA: United States Food and Drug Administration
EMA: European Medicines Agency
GvHD: graft-versus-host disease
GMP: good manufacturing practice
ICANS: immune effector cell-associated neurotoxicity syndrome
LV: lentivirus
MDSC: myeloid-derived suppressor cells
MHC: major histocompatibility complex
NK: natural killer
ScFv: single-chain variable fragment
SLE: systemic lupus erythematosus
TAM: tumor-associated macrophages
TCR: T cell receptor
TME: tumor microenvironment
Treg: regulatory T cell
VCN: vector copy number
VSV-G: vesicular stomatitis virus glycoprotein
VH: variable heavy chain
VL: variable light chain
CD28: cluster of differentiation 28
CD3ζ: cluster of differentiation 3 zeta subunit
CD19: cluster of differentiation 19
BCMA: B-cell maturation antigen
